# Risk Factors for Progression to Type 2 Diabetes in a Pediatric Prediabetes Clinic Population

**DOI:** 10.1210/jendso/bvad118

**Published:** 2023-10-12

**Authors:** Natasha Belsky, Jaclyn Tamaroff, Ashley H Shoemaker

**Affiliations:** Vanderbilt University School of Medicine, Nashville, TN 37212, USA; Division of Pediatric Endocrinology, Vanderbilt University Medical Center, Nashville, TN 37212, USA; Division of Pediatric Endocrinology, Vanderbilt University Medical Center, Nashville, TN 37212, USA

**Keywords:** prediabetes, type 2 diabetes, pediatric endocrinology

## Abstract

**Background:**

Pediatric type 2 diabetes (T2D) is increasing in prevalence, yet it is unclear what definition of pediatric prediabetes predicts progression to T2D. Strategies are needed to better identify at risk individuals who could benefit from early intervention.

**Methods:**

Retrospective chart review of a pediatric prediabetes clinic over 7 years. Inclusion criteria include hemoglobin A1c (HbA1C) and ≥1 glucose from oral glucose tolerance test. Exclusion criteria include type 1 diabetes, maturity onset diabetes of the young, or T2D on initial visit.

**Results:**

A total of 552 patients were included, 6.5% (n = 36) progressed to T2D over 2.4 ± 1.5 years. At initial visit, T2D progressors had a higher body mass index (38.6 ± 6.5 vs 34.2 ± 8.4 kg/m^2^, *P* = .002), HbA1C (6.0 ± 0.3%, vs 5.7± 0.3, *P* < .001), 2-hour glucose (141 ± 28 vs 114 ± 29 mg/dL, *P* < .001), and C-peptide (4.8 vs 3.6 ng/mL, *P* = .001). Fasting glucose was not significantly different. In a multivariable model, male sex (hazard ratio [HR], 2.4; *P* = .012), initial visit HbA1C (HR, 1.3 per 0.1% increase; *P* < .001), and 2-hour glucose level (HR, 1.2 per 10 mg/dL increase; *P* = .014) were all predictive of T2D progression. Patients who progressed to T2D had an increase in body mass index of 4.2 kg/m^2^ and children consistently taking metformin took longer to progress (43 ± 21 vs 26 ± 16 months; *P* = .016).

**Discussion:**

A total of 6.5% of patients with prediabetes developed T2D over a 7-year period. Initial visit laboratory values and weight trajectory may allow for risk stratification, whereas fasting plasma glucose is less helpful. Weight stabilization and metformin therapy could be important interventions for diabetes prevention in children.

The incidence and prevalence of type 2 diabetes mellitus (T2D) in youth has dramatically increased over the past 20 years, especially in racial and ethnic minority populations [1-3]. The SEARCH for Diabetes in Youth study estimates that the prevalence of youth-onset T2D will double to quadruple by 2050 [1-3]. One model estimated that the incidence of T2D in youth will increase 673% by 2060 [4]. Despite the rise in pediatric T2D, the pathophysiology and disease progression in children is not well understood. There are known differences in adult and pediatric β- cell responses [5] and insulin resistance can be a transient feature of puberty [6]. Prediabetes diagnostic criteria were derived from adult definitions based on long-term health outcomes and clinical trials in populations aged older than 18 years [7, 8]. It is thus not clear what definition of pediatric prediabetes predicts progression to T2D or long-term morbidity [9, 10].

Metformin is the initial treatment of choice for children and adolescents with T2D [11]. It is also standard of care for prevention of T2D in adults with prediabetes [12]. Metformin use in pediatric prediabetes is more controversial because it has not been clearly shown to decrease disease progression [5, 13]. The RISE Consortium data showed that pediatric patients with recent-onset T2D had continued deterioration of β-cell function despite metformin therapy [5]. Recent meta-analyses of metformin use in adolescents with obesity did find that metformin leads to a greater reduction in weight/body mass index (BMI) than placebo [13, 14]. In routine clinical practice, metformin is often used for prediabetes, insulin resistance, and weight management and is safe and well tolerated [14-16].

It is recognized that age of diagnosis is inversely associated with increased morbidity and mortality from T2D [17, 18]. Strategies are needed to better identify at risk children who could benefit from longitudinal follow-up and early intervention to slow or stop progression to T2D. Earlier diagnosis of T2D may also decrease initial treatment burden. To recognize children at risk of T2D, it is crucial to identify the factors that contribute to disease progression from prediabetes.

The Vanderbilt University Medical Center (VUMC) Pediatric Prediabetes Clinic [19] was founded in 2015, dedicated to patients referred to pediatric endocrinology for concerns of prediabetes and T2D. The goal of the clinic is to identify patients who could use more intensive monitoring and management. Patients all experience a uniform initial visit and are asked to return to follow-up at the prediabetes clinic if they have abnormal glucose tolerance or at the T2D clinic at the same site if they have T2D. Patients with normal glucose tolerance are prescribed lifestyle modification strategies and return to follow-up with their primary care physician. In this study, we conducted a chart review of patients evaluated in the VUMC Prediabetes Clinic over a 7-year period to determine risk factors for progression to T2D.

## Materials and Methods

### Study Population

The study protocol was approved by the Vanderbilt institutional review board. The study population included children ≤18 years of age who were seen at the VUMC Pediatric Prediabetes Clinic starting May 17, 2015, through August 30, 2022. Children were referred to the clinic for a documented abnormal blood glucose (fasting plasma glucose [FPG] ≥100 mg/dL, random glucose ≥150 mg/dL) or hemoglobin A1C (HbA1C) ≥5.9%. This HbA1c cutoff is higher than the typical definition of prediabetes (≥5.7%) but was chosen to enrich the clinic with higher risk patients because referral rates were greatly exceeding our clinic visit capacity. Children diagnosed with type 1 diabetes, maturity onset diabetes of the young, or T2D on initial visit were subsequently excluded from this analysis. Additionally, we excluded patients without a recorded point-of-care HbA1C and without at least 1 glucose result from the oral glucose tolerance test (OGTT).

For the initial visit, patients were classified either as normal glucose tolerance or impaired fasting glucose. Impaired fasting glucose criteria were fasting glucose of 100 to 125 mg/dL and/or 2-hour glucose of 140 to 199 mg/dL [20]. Based on follow-up visits, patients were assigned to 1 of 2 cohorts for further analysis: T2D progression or nonprogression. Group assignments were based on the 2022 American Diabetes Association criteria: FPG ≥126 mg/dL, 2-hour plasma glucose ≥200 mg/dL, or HbA1C ≥ 6.5% [20]. In the absence of unequivocal hyperglycemia, the diagnosis requires 2 abnormal results; 1 abnormal result is acceptable if symptoms of diabetes are present.

### Clinic Protocol

The VUMC Pediatric Prediabetes Clinic was founded in 2015 to evaluate patients younger than age 18 years referred by a primary care pediatrician for concern for prediabetes or T2D. Patients are scheduled for a morning visit, during which they undergo fasting laboratory testing, including a 2-hour 75-g OGTT, lipid panel, C-peptide, and complete metabolic panel. HbA1C is also measured on a point-of-care machine. All clinic patients were required to have a laboratory HbA1C (by high-performance liquid chromatography) if a previous HbA1C by high-performance liquid chromatography was unavailable to screen for hemoglobinopathies. For analysis, the point-of-care HbA1C was used. Height was measured without shoes using a wall-mounted stadiometer, weight was measured using a digital scale, and blood pressure was measured with an automatic cuff. Patients were seen by a pediatric endocrinologist for evaluation of diabetes risk, a registered dietician for dietary counseling, and a specially trained general pediatrician or pediatric nurse practitioner for motivational interviewing around lifestyle change. Patients with an OGTT consistent with T2D, and most patients with an OGTT consistent with impaired glucose tolerance were prescribed metformin 2000 mg/day and scheduled for every-3-month follow-up with a diabetes provider and registered dietitian.

### Data Collection

Data were collected via retrospective chart review of the electronic medical record and stored in a deidentified REDCap database [21]. Study personnel used the Clinical Data Interoperability Services tool to enable transfer of information from the electronic health record into REDCap. Demographic information collected on each patient included age, gender, race, and ethnicity. Clinical data were collected from each clinic visit including blood pressure, weight, BMI, and clinical laboratory values (point-of-care HbA1C, C-peptide, 2-hour glucose, FPG, lipid panel, aspartate aminotransferase, alanine transaminase).

For those with T2D progression, a full manual chart review was completed to validate that each T2D progression case was true and to capture additional information. For these patients, time from first visit to diagnosis of T2D was calculated. Other information such as family history of T2D, insurance type, time since last contact with the Vanderbilt system, travel distance to the clinic, symptoms of diabetes, presence of acanthosis, current medications, pubertal status, and comorbid conditions (polycystic ovary syndrome, asthma, obstructive sleep apnea) were noted. Additionally, any intervention for the patient at each clinic visit was noted including diet and exercise modification, medication prescription, or a combination. At each subsequent clinic visit, medication compliance was evaluated using patient recall and noted as either taking as prescribed, missing doses, or not taking medication. If the patient was not taking the full prescribed dose of the medication, this was classified under “missing doses.”

### Data Analysis

Statistical analyses were performed using SPSS version 28. All data are presented as mean ± SD or as percentages. Baseline continuous variables were analyzed by Student *t* test. If Levene's test for equality of variances was significant, equal variances were not assumed. Categorical variables were analyzed by Fisher exact test or χ^2^ test. Cox regression with multivariable analysis was used to assess relationship between covariates and time to the event. *P* < .05 was considered statistically significant.

## Results

### Study Population

A total of 678 charts were reviewed for study inclusion. Patients were excluded for diagnosis of type 1 diabetes (n = 14), monogenic diabetes (n = 1), T2D at initial visit (n = 62), and missing laboratory data (n = 49) at the first clinic visit. Of the remaining 552 patients included in the study, 36 (6.5%) progressed to T2D during the duration of the study period. Per the clinic protocol [19], patients without impaired glucose tolerance received follow-up care at their primary care office, but 315 of 516 patients in the nonprogression group had subsequent HbA1c data available in our medical record system to confirm their group status. Baseline demographic characteristics are shown in Table 1. The nonprogression and T2D progression groups were similar in age at the initial visit (13.0 ± 2.8 vs 12.1 ± 2.2 years; *P* = .06). Race and ethnicity were not different between groups. The T2D progression group had a greater degree of obesity, with an average BMI 4.4 kg/m^2^ higher than the nonprogression group (95% CI, 1.6-7.2 kg/m^2^; *P* = .002).

**Table 1.** Baseline characteristics and laboratory Findings

	T2D nonprogression (n = 516)	T2D progression (n = 36)	*P* value
Age (y)	13.0 ± 2.8	12.1 ± 2.2	.06
Sex (%)			.06
Female	56.6	38.9	
Male	43.4	61.1	
Ethnicity (%)			.63
Hispanic	13.2	11.1	
Non-Hispanic	61.0	77.8	
Race (%)			.18
White	35.4	33.3	
Black	30.4	36.1	
Asian	1.7	0	
Multiple races	3.5	11.1	
Other	2.9	0	
Unknown	26.0	19.4	
Height (cm)	158.8 ± 13.1	162.1 ± 12.3	.15
Weight (kg)	88.2 ± 28.9	102.4 ± 22.8	.004
BMI (kg/m^2^)	34.2 ± 8.4	38.6 ± 6.5	.002
SBP (mm Hg)	130 ± 12	132 ± 10	.39
DBP (mm Hg)	72 ± 8	72 ± 9	.80
HbA1C (%)	5.7 ± 0.3	6.0 ± 0.3	<.001
Fasting C-peptide (ng/mL)	3.6 ± 2.0	4.8 ± 2.1	.001
2-hour glucose (mg/dL)	114 ± 28	141 ± 29	<.001
Fasting glucose (mg/dL)	92 ± 9	94 ± 13	.26
LDL	94 ± 27	94 ± 32	.95
Triglycerides	109 ± 59	138 ± 64	.02
HDL	41 ± 9	38 ± 5	.003
Total cholesterol	157 ± 31	159 ± 32	.78
ALT	31 ± 31	44 ± 43	.17
AST	28 ± 17	32 ± 22	.33

Results presented as percent or mean ± SD. Number of subjects (T2D nonprogression group, TD progression group): ethnicity (n = 382, n = 32), SBP and DBP (n = 502, n = 36), fasting C-peptide (n = 471, n = 32), 2-hour OGTT (n = 511, n = 36), fasting glucose (n = 513, n = 36), LDL (n = 346, n = 27), triglycerides (n = 347, n = 27), HDL (n = 347, n = 27), total cholesterol (n = 346, n = 27), and ALT (n = 329, n = 26), AST (n = 329, n = 26). Continuous variables were analyzed by 2-way *t* test and categorical variables were analyzed by Fisher exact test (sex, ethnicity) and χ^2^ (race).

Abbreviations: ALT, alanine aminotransferase; AST, aspartate transaminase; BMI, body mass index; DBP, diastolic blood pressure; HbA1C, hemoglobin A1c; OGTT, oral glucose tolerance test; SBP, systolic blood pressure; T2D, type 2 diabetes.

### Diabetes Laboratory Markers

**Figure 1.**
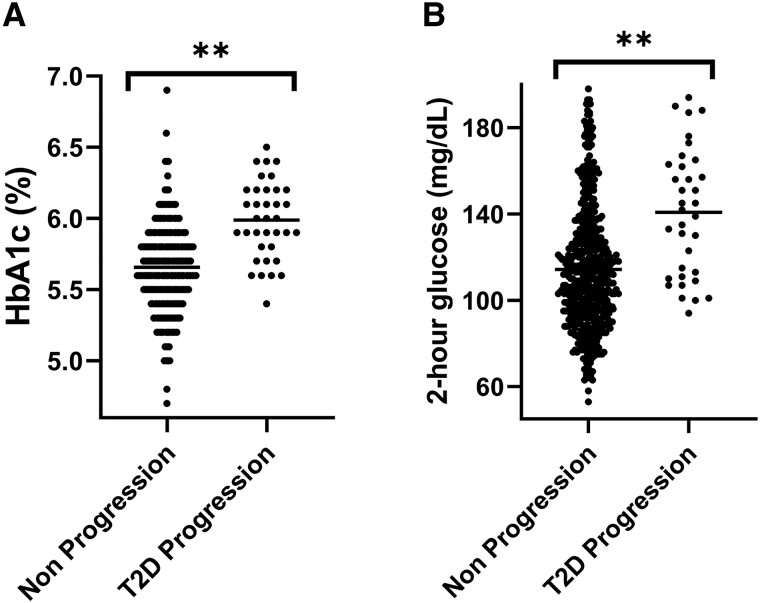
Initial HbA1C (A) and 2-hour glucose (B) in the type 2 diabetes (T2D) progression and nonprogression groups; **P < .001 for the difference in each parameter.

Baseline laboratory findings are reported in Table 1. Initial visit HbA1C (mean difference, 0.3%; 95% CI, 0.2-0.4; *P* < .001), fasting C-peptide (mean difference, 1.2 ng/mL; 95% CI, 0.5-1.9 ng/mL; *P* = .001), and 2-hour glucose (mean difference, 27 mg/dL; 95% CI, 17-36 mg/dL; *P* < .001) were significantly higher in the T2D progression group (Fig. 1). There was no difference in fasting glucose between groups (mean difference, 2 mg/dL; 95% CI, −2 to 7 mg/dL; *P* = .26).

### T2D Progression

**Figure 2.**
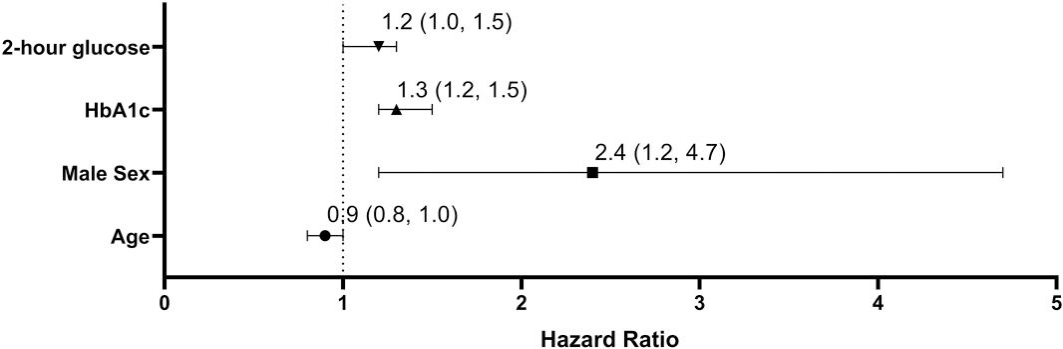
Multivariate analysis of progression from prediabetes to type 2 diabetes (T2D). *P* < .01 for 2-hour glucose, HbA1C, and male sex. Hazard ratios of progression (95% CI) are presented per 10 mg/dL change in 2-hour glucose, per 1% change in HbA1C and per 1 year of age.

In a multivariable model (Fig. 2), male sex (hazard ratio [HR], 2.4; 95% CI, 1.2-4.7; *P* = .01), every 0.1% increase in HbA1C (HR, 1.3; 95% CI, 1.2-1.5; *P* < .001) and every 10-mg/dL increase in 2-hour glucose (HR, 1.2; 95% CI, 1.0-1.3; *P* = .01) were all associated with increased risk of progression to T2D. Age was also included in the model but did not reach statistical significance (HR, 0.9; 95% CI, 0.8-1.0; *P* = .05).

**Figure 3.**
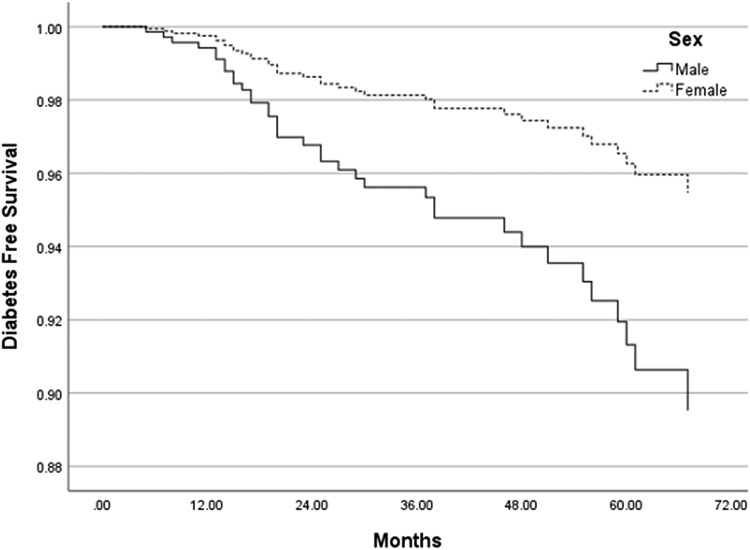
Diabetes-free survival analysis of the progression from prediabetes to type 2 diabetes (T2D) in males and females.

On average, it took 28.8 ± 17.9 months for patients to progress to diabetes after their initial visit. The average age at T2D diagnosis was 14.9 years, but it ranged from 9.9 to 18.3 years. Males progressed at a greater rate than females (Fig. 3). Of patients who progressed, 94% had a family history of T2D, 92% had public insurance, 94% had acanthosis at their initial visit, and 72% had impaired glucose tolerance at their initial visit. 58% of patients were diagnosed at their second clinic visit. Most patients (89%) who progressed to T2D had an increasing BMI from initial visit to diagnosis. Compared with baseline, BMI increased an average of 4.2 ± 4.3 kg/m^2^ from onset to diagnosis. Patients were not started on any glucagon-like peptide-1 receptor agonists (GLP1RA) or other glucose-lowering therapy except metformin before diagnosis. At T2D diagnosis, 6 patients required insulin, 1 was prescribed a GLP1RA, and the remaining 28 patients were treated with metformin monotherapy. The average A1c at diagnosis was 8.1%, ranging from 6.5% to 13.4%. Approximately half (53%) of the patients had an HbA1C <7% at diagnosis.

Of the 36 patients who progressed, 23 were prescribed metformin at their initial clinic visit. At the time of T2D diagnosis, 6 patients were taking metformin as prescribed (2000 mg per day), 9 were missing some doses or not taking the fully prescribed dose, and 8 were not taking the medication at all. Patients who were taking metformin as prescribed had the lowest average HbA1C (7.1 ± 0.6%) at diagnosis and took 17 months longer to progress to T2D compared with the other groups combined (43 ± 21 vs 26 ± 16 months; *P* = .016; Table 2).

**Table 2.** Time to T2D diagnosis and HbA1c by metformin prescribing practices and medication compliance

	Prescribed metformin, taking (n = 6)	Prescribed metformin, missing doses (n = 9)	Prescribed metformin, not taking (n = 8)	Not prescribed metformin (n = 13)
Average HbA1c (%)	7.1 ± 0.6	7.1 ± 0.9	10.9 ± 2.6	7.7 ± 2.0
Average time to diagnosis (months)	43 ± 21	20 ± 15	28 ± 15	29 ± 18

Abbreviations: HbA1C, hemoglobin A1C; T2D, type 2 diabetes.

## Discussion

Over the past 3 decades, there has been a sharp increase in the incidence and prevalence of childhood obesity, prediabetes, and T2D; at least 1 in 5 adolescents are estimated to have prediabetes [22-24]. Well-established determinants predicting the natural history of prediabetes in youth is lacking because definitions and screening recommendations are based on long-term health outcomes in adults (American Diabetes Association). Thus, there is a critical need for a longitudinal follow-up of at-risk youth to allow us to identify and target patients for prevention and early intervention. Further characterization of diabetes pathophysiology could help primary care physicians and endocrinologists manage and stratify risk in the large number of patients screening positive for prediabetes. This report describes a large, multiethnic, clinical cohort from the United States with long-term follow-up of children and adolescents with prediabetes.

During our 7-year study period, only 6.5% of patients progressed from prediabetes to T2D. This is consistent with observations from previously published, smaller cohorts that have reported 1% to 8% progression over a 1- to 3-year follow up [25-27]. There is 1 published study reporting higher rates of progression in a group of patients who did not comply with nutrition follow-up; 22.6% progressed to T2D over a median of 30 months (n = 66) [28, 29]. That study did not provide information on criteria used for diagnosis of T2D or HbA1C values for patients diagnosed with T2D. It is possible that less rigorous criteria were used, such as a single HbA1C ≥6.5%, leading to a higher rate of T2D diagnosis.

Our standard protocol for baseline evaluation in the Vanderbilt Prediabetes Clinic allowed us to examine numerous risk factors for progression to T2D. We found higher baseline HbA1C, fasting C-peptide, and 2-hour glucose to be associated with progression to T2D. In our multivariable analysis, both HbA1C and the 2-hour glucose were strong independent predictors of progression. Importantly, FPG was not associated with progression to T2D, similar to previous findings from a smaller cohort [30]. There is debate in pediatrics about how to best screen for T2D risk [9, 31]. Our group and others have showed that HbA1C, FPG, and 2-hour glucose are poorly correlated to each other and measure different underlying pathology [19, 32]. Impaired glucose tolerance may be associated with more severe insulin resistance and reduced insulin secretion [32]. Elevations in these measures can be transient, particularly in adolescents, in whom puberty can lead to insulin resistance [25, 27, 30, 33, 34]. The presence of more than 1 glucose abnormality may better predict risk of T2D progression [25, 32]. Based on our data, HbA1C plus a nonfasting glucose may be a feasible way to identify high-risk pediatric patients in a clinical setting.

In addition to laboratory measures, worsening obesity is strongly associated with T2D progression [35]. In our cohort, patients who progressed to T2D had a higher BMI at baseline and saw continued gains with an average increase of 4.2 kg/m^2^ from initial visit to T2D diagnosis, consistent with previous studies [19, 26, 30]. The Diabetes Prevention Program demonstrated that in adults, weight loss was the main predictor of reduced T2D incidence [36]. In pediatric patients, stabilizing weight may also reduce T2D risk. There are now multiple antiobesity medications approved in children 12 years and older. Although these medications have not been evaluated for treatment of prediabetes, the data suggest that treatment of obesity may be an important step for prevention of progression to T2D.

Historically, population-based studies have described a higher incidence and prevalence of T2D in females compared with males [37, 38]. In our cohort, although more females were referred to the clinic because of prediabetes (55.4%), males progressed more commonly and more quickly than females. A recent nationwide study described that during the pandemic, there was an increase in proportion of adolescent male youths diagnosed with T2D [38]. It is unclear why we saw a higher percentage of T2D in males, but perhaps because of the historical lower prevalence of T2D in males and less societal stigma surrounding male obesity, males were farther along in disease progression by the time of the Prediabetes Clinic referral. In addition, this male predominance fits with the previously described demographics of our clinic population. In a recent review of 100 patients diagnosed with T2D at the Vanderbilt Eskind Pediatric Diabetes Clinic, 55% were male.

Nationwide data have identified profound racial and ethnic disparities in the incidence and prevalence of T2D in youth [30, 39]. The highest burden of diabetes is in American Indian and non-Hispanic Black (NHB) youth [1]. Our study showed no significance in differences in the progressor vs nonprogressor groups based on race or ethnicity. Our Prediabetes Clinic population is enriched for NHB patients. Vanderbilt resides in Davidson County, which is 27.4% NHB, and the state of Tennessee is 17.1% NHB (census.gov population estimates July 1, 2021). Our overall Prediabetes Clinic population was 30.8% and the T2D progressor group was slightly, though not significantly, higher at 36.1%. Because our data were collected retrospectively, we were limited by data available in the medical record. There was a high degree of missing data for ethnicity (24.8%) and race (25.5%), which may have limited our ability to detect its role in T2D progression risk.

Importantly, patients evaluated in the Prediabetes Clinic were less likely to present with severe T2D than other patients with new-onset T2D at Vanderbilt and in published data. In a national cohort, 9% (prepandemic) and 20% (2020-2021, during the COVID-19 pandemic) presented in diabetic ketoacidosis with an average HbA1C >9% [40]. A chart review of the most recent 100 patients diagnosed with new-onset T2D at our center found that 54% presented with an HbA1C >8.5%, the commonly accepted threshold for starting insulin [11]. In contrast, the average HbA1C of T2D progressors in this smaller cohort evaluated in the Prediabetes Clinic was 8.1%; only 1 patient (3%) presented with diabetic ketoacidosis, 6 patients (17%) required insulin, and 1 patient was prescribed a GLP1RA at diagnosis. It is possible that the clinic model of identifying high-risk patients, counseling families on diabetes risk and symptoms of diabetes, and engaging primary care physicians for future monitoring contributed to earlier diagnosis.

There is debate surrounding metformin's role in diabetes prevention in children [41, 42]. Clinical trials in the prediabetes population, some of which have included children, have indicated the metformin may help delay progression to diabetes, pointing to the improvement in BMI and body composition [43-46]. In this cohort, the average time to diagnosis was far longer in patients consistently taking metformin (43 months), in comparison to those not taking the prescribed medication (28 months) or never prescribed metformin (29 months). HbA1C at diagnosis was also lower in patients taking metformin. Patients taking their medication as prescribed may also have been making more impactful lifestyle changes or have had more regular follow-up for prediabetes management. Metformin monotherapy for T2D has higher failure rates in NHB adolescents [47]. Although not statistically significant, a greater percentage of NHB progressed to T2D during this study period. Further research is needed to determine if metformin use may help delay progression to T2D and whether there may be racial differences.

There are various limitations to this study, including missing data. It is known that patients with prediabetes and T2D have a high risk of loss to follow-up. A previous study of a multicenter US cohort found that 55% of T2D patients are lost to follow-up after a median of 1.3 years [48]. It is possible that additional Prediabetes Clinic patients developed T2D during the study period without our knowledge because we did not contact patients to confirm their disease status or require clinic follow-up to be included in the cohort. This is most likely in patients who became adults during the study period and could have sought care outside of our practice. It is less likely in the pediatric patients because most primary care providers in our area are uncomfortable managing prediabetes and T2D and there is a lack of competing pediatric endocrinology practices in our region. Thus, although patients with alternate providers or those who never returned to clinic may have progressed to T2D without our knowledge, this is highly unlikely. Last, the COVID-19 pandemic may have impacted our results as follow up, lifestyle habits, and overall rates of progression to diabetes were all affected by the pandemic.

In summary, only 6.5% of pediatric patients with prediabetes developed T2D over the 7-year period of our study, highlighting the importance of identifying which patients would most benefit from intensive lifestyle interventions or antidiabetes and antiobesity medications. Initial visit laboratory values, particularly HbA1C and nonfasting glucose, along with worsening obesity, may allow for risk stratification. Fasting plasma glucose is less helpful. Preventing further worsening of obesity is an important intervention for T2D prevention in children and metformin may have a role in the management of pediatric prediabetes.

## Data Availability

Some or all datasets generated during and/or analyzed during the current study are not publicly available but are available from the corresponding author on reasonable request.

## Abbreviations

BMI: body mass index
FPG: fasting plasma glucose
GLP1RA: fasting plasma glucose
HbA1C: hemoglobin A1C
HR: hazard ratio
NHB: non-Hispanic Black
OGTT: oral glucose tolerance test
T2D: type 2 diabetes
VUMC: Vanderbilt University Medical Center
